# Acute Ischemic Stroke as the Initial Manifestation of Antiphospholipid Syndrome After SARS-CoV-2 Infection in a 17-Year-Old Patient

**DOI:** 10.7759/cureus.103286

**Published:** 2026-02-09

**Authors:** Carlos A Portales Cameras, Mauricio E Rodríguez Vergara, Carlos A Gómez Núñez, Daniel E Córdova Galván, Alberto Cervantes-Hernández

**Affiliations:** 1 Internal Medicine, Hospital Angeles del Pedregal, Mexico City, MEX; 2 Internal Medicine, Hospital Angeles del Pedregal, Mexico city, MEX; 3 Radiology, Hospital Angeles del Pedregal, Mexico City, MEX; 4 Medicine, Facultad Mexicana de Medicina, Universidad La Salle, Mexico City, MEX

**Keywords:** antiphospholid antibody syndrome, antiphospholipid antibody, covid-19, ischemic stroke, rescue mechanical thrombolysis

## Abstract

Stroke is an unusual condition in young adults, but when it presents in this population, there are specific causes that differ from those in older adults. When this occurs, it is necessary to make an extensive assessment to establish a final diagnosis. One of these causes, as in the case presented, is antiphospholipid syndrome (APS), which can present as venous or arterial thrombosis. There are conditions that act as triggers for the formation of antiphospholipid antibodies (aPL) and the development of APS. This report illustrates these points by presenting a young patient who developed APS after a severe acute respiratory syndrome coronavirus 2 (SARS-CoV-2) infection that acted as a trigger, along with probable genetic susceptibility factors, and was diagnosed in accordance with guidelines 12 weeks after initial presentation. He was successfully treated with rescue mechanical thrombolysis, with favorable outcomes.

## Introduction

Stroke is a common condition in older adults; however, only 15 to 18% of ischemic strokes occur in young adults. The incidence of ischemic stroke in this population ranges from seven to 100 cases per 100,000 person-years [1]. The causes of ischemic stroke in young adults differ from those in older adults, although the age cutoff defining “young adults” is variable. In some studies, it is considered to be younger than 40 years, while others extend it to younger than 60 years. The etiology of ischemic stroke is usually clearer in older adults than in young patients. In young adults, embolic sources such as atrial myxoma, dilated cardiomyopathy, intracardiac thrombus, atrial septal defect, nonbacterial thrombotic endocarditis, patent foramen ovale, and left ventricular hypokinesis can cause ischemic stroke. Additionally, other less common causes, including reversible cerebral vasoconstriction syndrome, Moyamoya disease, arterial dissection, cerebral venous thrombosis, antiphospholipid syndrome (APS), and protein S deficiency, are more frequently observed in young adults [1].

Among these causes, APS accounts for approximately 7-15% of ischemic strokes in young adults, reflecting its prothrombotic nature [2]. The impact of stroke on quality of life is greater in young adults than in older individuals due to their longer life expectancy [1]. APS is a systemic autoimmune disease characterized by the persistent presence of elevated antiphospholipid antibodies (aPL) and recurrent arterial and/or venous thrombosis, as well as obstetric morbidity. It can be classified as primary, when it occurs as an isolated condition, or secondary, when it is associated with another autoimmune disease, most commonly systemic lupus erythematosus (SLE) [3]. APS is a recognized cause of acute ischemic stroke and transient ischemic attack (TIA) in young adults. In individuals younger than 50 years, 17% of strokes and 12% of TIAs have been associated with APS, with some reports indicating rates of up to 20% in patients under 45 years of age. Thrombosis leading to the occlusion of large intracranial arteries, such as the middle cerebral artery (MCA), represents the main pathophysiological mechanism [2,3].

Ischemic strokes reported in the context of coronavirus disease 2019 (COVID-19) infection have been associated with a hypercoagulable state and elevated D-dimer and C-reactive protein levels. However, a causal relationship has not been established, and although there are data addressing the association between COVID-19 and the first thrombotic event in APS and its pathophysiology, the evidence remains limited [4].

## Case presentation

A 17-year-old male presented to the emergency department after awakening with dysarthria, right facial paralysis, and proportional right hemiparesis. These symptoms had occurred six hours after the last time he had been observed in his baseline neurological state. On admission, the neurological examination revealed dysarthria; cranial nerve VII with right central facial paralysis; right hemibody weakness with proximal and distal strength of 1/5 on the Daniels Scale; muscular stretch reflexes +/++++; ataxia; and an extensor plantar response.

His medical history included Kawasaki disease at five years of age, which was treated with intravenous immunoglobulin. He denied alcohol, tobacco, or other drug use. He had been diagnosed with acne vulgaris and treated with isotretinoin for the past eight months. Family history was significant for his paternal grandmother, who died at 42 years of age from an unspecified thrombotic event; a paternal uncle who experienced a pulmonary embolism at 33 years of age and was a carrier of the factor II mutation (G20210A); and his mother, who had urticaria of autoimmune origin. Four weeks before admission, he had an upper respiratory tract infection caused by severe acute respiratory syndrome coronavirus 2 (SARS-CoV-2).

**Figure 1:**
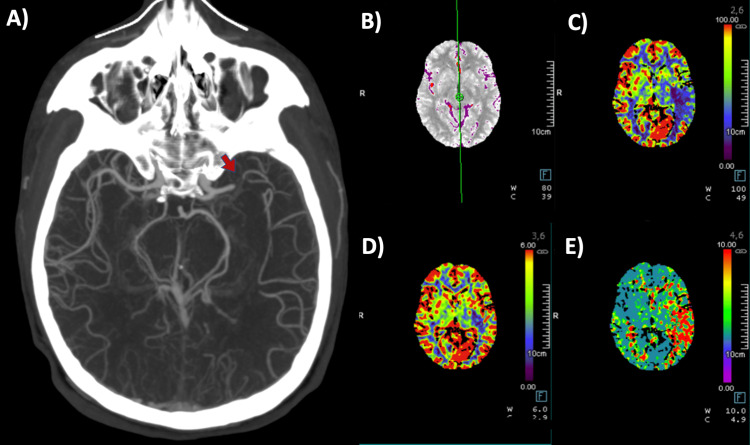
Brain CT angiography images The images demonstrate left MCA occlusion in the M1 segment (arrow) (A); reference perfusion processing (B), with hypoperfusion area with low blood flow (C); and low blood volume (D); and an increase in median transit time (E) in cerebral perfusion maps CT: computed tomography; MCA: middle cerebral artery

Laboratory evaluation, including complete blood count, creatinine, liver function tests, electrolytes, lipid profile, urinalysis, and C-reactive protein, was within normal limits. A brain CT angiography demonstrated occlusion of the left MCA in the M1 segment (Figure 1).

**Figure 2:**
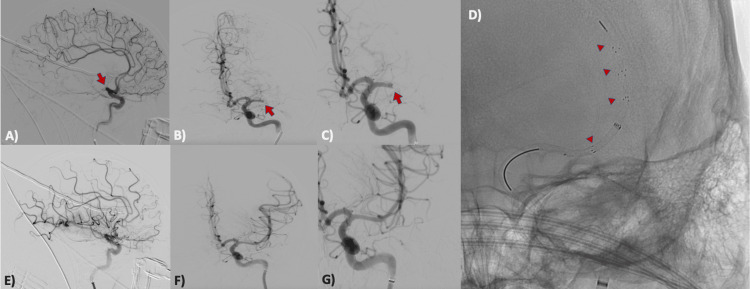
Cerebral angiography images Anterior circulation arteriography demonstrating occlusion of the left MCA in its M1 segment (arrow) in lateral (A), AP (B), and magnified AP (C) projections. (D) AP projection during mechanical thrombectomy procedure, with stent-retriever (arrowheads) in MCA. Posterior control projections (E, F, and G) with complete recanalization of the vessel (TICI 3) MCA: middle cerebral artery

On the day of the patient's admission, cerebral angiography and rescue mechanical thrombectomy were performed (Figure 2; Videos 1-4).

**Video 1:** Dynamic arteriography of anterior circulation prior to thrombectomy in AP projection showing occlusion of the MCA in its M1 segment MCA: middle cerebral artery

**Video 2:** Dynamic arteriography of anterior circulation prior to thrombectomy in lateral projection, showing occlusion of the MCA in its M1 segment MCA: middle cerebral artery

**Video 3:** Dynamic arteriography of anterior circulation after thrombectomy in AP projection, with complete recanalization of the vessel (TICI 3)

**Video 4:** Dynamic arteriography of anterior circulation after thrombectomy in lateral projection, with complete recanalization of the vessel (TICI 3)

The patient was admitted to the ICU. Two days later, acetylsalicylic acid 100 mg once daily and enoxaparin 40 mg once daily were initiated. The evaluation for secondary causes of thrombosis was performed immediately after confirmation of ischemic stroke by CT angiotomography. Initial laboratory testing included antithrombin III, protein S, protein C, and homocysteine, all of which were within normal ranges. A borderline lupus anticoagulant (LAC) was detected, with borderline values for the ratio dRVVT screen, LAC ratio dRVVT confirm, and LAC ratio normalized dRVVT. IgM and IgG anticardiolipin (aCL) antibodies and IgM and IgG anti-beta 2 glycoprotein 1 (aβ2GP1) antibodies were negative.

Based on these results, further evaluation for additional secondary causes of thrombosis was performed, including factor V (factor V Leiden mutation), prothrombin 20210 G-A mutation, JAK2 V617F mutation, paroxysmal nocturnal hemoglobinuria (PNH), and CD phenotypes. All tests were negative. Finally, MTHFR 667C heterozygote p.[Ala222Val];[222=] was identified; however, this finding was considered clinically insignificant. The results and their reference values are shown in Table 1. 

**Table 1:** Initial laboratory results for secondary causes of thrombosis LAC: lupus anticoagulant; aCL: anticardiolipin antibodies; aβ2GP1: anti-beta 2 glycoprotein 1 antibodies; PNH: paroxysmal nocturnal hemoglobinuria

Laboratory test	Result	Reference range
Antithrombin III	123.00%	75.00-125.00%
Protein S	89.60%	61.00-145.00%
Protein C	83.00%	69.00-134.00%
Homocysteine	7.15 umol/L	5.40-16.20 µmol/L
LAC ratio dRVVT screen	1.42	0.00-1.20
LAC ratio dRVVT confirm	1.01	0.00-1.20
LAC ratio normalized dRVVT	1.41	0.00-1.20
IgM aCL antibody	1.30 U/mL	< 20.00 U/mL
IgG aCL antibody	3.40 U/mL	< 20.00 U/mL
IgM a β2GP1 antibody	0.30 U/mL	< 20.00 U/mL
IgG a β2GP1 antibody	13.40 U/mL	< 20.00 U/mL
Factor V (factor V Leiden mutation)	Not detected	Not detected
Prothrombin 20210 G-A mutation	Not detected	Not detected (<2ng/uL)
JAK2 V617F mutation	Not detected	Not detected
PNH CD phenotypes	Not detected	Not detected
MTHFR 667C mutation	Heterozygote p.[Ala222Val];[222=]	Not detected

**Figure 3:**
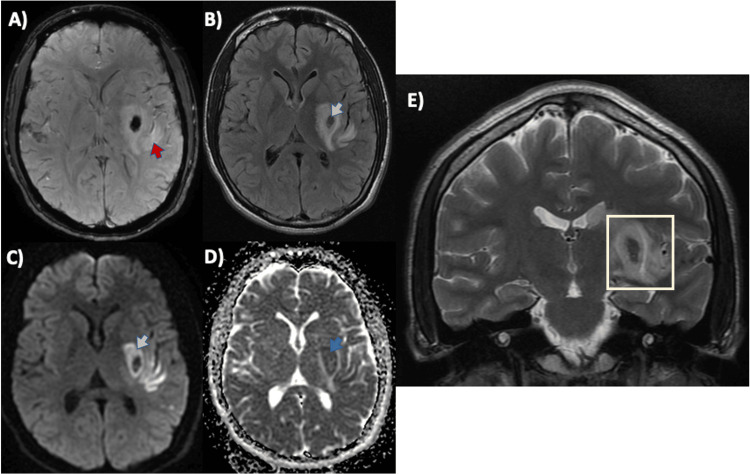
Brain MRI findings A) FLAIR sequence showing an area of peri-infarct edema (red arrow) and (B) magnetic susceptibility sequence demonstrating H2-type hemorrhagic transformation (ECASS), involving <30% of the infarct area (orange arrow). C, D) Diffusion (green arrow) and ADC (blue arrow) map showing diffusion restriction in the left lenticulostriate territory. E) T2 sequence delineating the affected left lenticulostriate region (yellow square) MRI: magnetic resonance imaging; FLAIR: fluid-attenuated inversion recovery; ADC: apparent diffusion coefficient

Vertebral and carotid Doppler ultrasonography, as well as 24-hour Holter monitoring and transthoracic echocardiography, were performed and found to be within normal limits. The patient showed progressive neurological recovery, with improvement of right facial paralysis, which evolved into facial palsy. He also improved in the ipsilateral hemibody paresis, mainly in the upper extremity, with muscle strength improving to 3/5. On the fourth day after angiography, a follow-up MRI showed hemorrhagic transformation (Figure 3).

However, the patient did not develop new neurological signs or symptoms. Given his clinical recovery at that time, he was discharged two days later without further complications. Twelve weeks later, aPL testing was repeated and showed positive results for IgG aCL of 40.10 U/mL, representing moderate titers, and IgG aβ2GP1 in 24.0 U/mL, representing low titers. LAC remained borderline with: ratio dRVVT screen 1.26 (0.0-1.20), ratio dRVVT confirm 0.93 (0.0-1.20), ratio normalized dRVVT 1.35 (0.0-1.20) (Table 2). Based on these findings, anticoagulation with acenocumarol (more readily accessible than warfarin) was initiated.

**Table 2:** Antiphospholipid antibodies (aPL) results after 12 weeks LAC: lupus anticoagulant; aCL: anticardiolipin antibodies; aβ2GP1: anti-beta 2 glycoprotein 1 antibodies

Laboratory test	Result	Reference range
LAC ratio dRVVT screen	1.26	0.00-1.20
LAC ratio dRVVT confirm	0.93	0.00-1.20
LAC ratio normalized dRVVT	1.35	0.00-1.20
IgM aCL antibody	6.30 U/mL	< 20.00 U/mL
IgG aCL antibody	40.10 U/mL	< 20.00 U/mL
IgM aβ2GP1 antibody	1.00 U/mL	< 20.00 U/mL
IgG aβ2GP1 antibody	24.00 U/mL	< 20.00 U/mL

## Discussion

APS is a clinically significant entity characterized by arterial and/or venous thrombotic complications. This case illustrates a significant example of the phenotypic variability seen in patients with APS. It highlights an APS presentation in a very young male patient, whereas most cases occur in females around 50 years of age. Although APS is more prevalent in females, primary thrombotic APS exhibits a more balanced sex distribution. It is often diagnosed in young adults; however, the mean age of diagnosis is around 50 years [2]. Approximately 50% of these patients have primary APS, while the remaining 50% have secondary APS [3].

APS is one of the leading causes of stroke in young adults and should always be considered in this population [1]. As part of the evaluation of ischemic stroke in a young patient without cardiovascular risk factors, an extensive work-up was performed. All investigations were negative, except for the presence of antiphospholipid antibodies (aPL). Other potential causes were excluded, including the MTHFR 667C heterozygous variant, which has no clinical significance. The pathophysiology of APS involves three key components: the coagulation cascade, endothelial cells, and immune cells. Antiphospholipid antibodies, produced by B-cells, are directed against specific phospholipid-binding proteins. Their interaction activates endothelial cells, upregulates pro-coagulant factors, and stimulates inflammatory processes [3].

Thrombotic events in APS are explained by the two-hit hypothesis, which also clarifies why thrombosis occurs intermittently despite the presence of aPL. The first hit creates a prothrombotic environment by disrupting the endothelium and its function, thereby promoting hypercoagulability. This first hit includes the presence of aPL and increased oxidative stress. Genetic predisposition, individual susceptibility, and hereditary factors play an important role, as susceptible individuals may develop aPL in response to an infection or autoimmune disease. These susceptibility factors themselves may act as the first hit, even in the absence of pre-existing aPL, coinciding with the eventual development of aPL. Thrombosis then occurs when there is another prothrombotic or endothelial-damaging condition-the second hit-which represents a stressor, such as trauma, surgery, or infection. This second insult promotes thrombus formation [3,5-7].

A stronger association with thrombotic events has been demonstrated for IgG aPL compared with IgM aPL. It has been concluded that IgM may play a more limited role, serving primarily a prognostic rather than a diagnostic function [2]. More significant thrombotic complications have been observed with the IgG isotype than with the IgM isotype [8]. Since the emergence of COVID-19, several reports and studies have described the development of APS after COVID-19 infection. COVID-19 may amplify inflammatory pathways and exacerbate prothrombotic mechanisms. Consequently, COVID-19 has been associated with APS, acting as a potential trigger in susceptible patients [9].

This case supports the concept that infection may act as a trigger for APS, specifically COVID-19, leading to immune system dysregulation and activation of alternative immunological pathways. The patient had an underlying genetic susceptibility/predisposition, representing the first hit, and COVID-19 infection, considered the second hit, which led to the development of aPL and a prothrombotic state. This case also demonstrates seroconversion of aPL in the post-COVID-19 infection period. Initially, no aPL were detected, with only a borderline LAC. Upon reassessment 12 weeks later, as recommended by current guidelines, IgG aCL antibodies and IgG aβ2GP1 antibodies were identified. This reflects the development of aPL after the clinical event (in this case, stroke) and confirms the diagnosis.

Traditionally, APS was diagnosed using the Sapporo criteria, which were later revised at the Sydney International Antiphospholipid Antibodies Congress [3]. In 2023, the American College of Rheumatology (ACR) and the European Alliance of Associations for Rheumatology (EULAR) established new classification criteria [3,9]. For appropriate diagnosis and classification, determination of the three antiphospholipid antibodies is required: LAC, IgM and IgG aCL antibodies, and IgM and IgG aβ2GP1 antibodies. After the initial assessment, it is necessary to check for persistence and/or positivity at least 12 weeks after the clinical event. This step is essential to exclude false-positive results associated with some bacterial and viral infections [2].

The entry criteria according to ACR/EULAR include at least one documented clinical criterion and a positive antiphospholipid antibody test, followed by accumulation of clinical and laboratory points according to the different domains. APS is classified if there are at least 3 points from clinical domains and 3 points from laboratory domains. It is important to emphasize that these are classification criteria, not diagnostic criteria [10]. Our patient met the entry criteria, with the specific domains adding up to 8 points-4 clinical points corresponding to arterial thrombosis in a patient without cardiovascular risk factors, and 4 laboratory points for moderate IgG aCL titers. This satisfies the requirement of at least 3 points from both clinical and laboratory domains. Based on the clinical condition, imaging findings, and time of symptom onset, the patient was successfully managed with rescue mechanical thrombectomy, resulting in progressive neurological recovery.

Management of APS must be multidisciplinary, ensuring continuous monitoring to prevent complications. For secondary prevention, current guidelines recommend long-term administration of vitamin K antagonists with a target INR of 2.0-3.0. There is insufficient evidence for the use of direct oral anticoagulants [2-3].

## Conclusions

APS is one of the most important causes of ischemic stroke in young adults. It is essential to rule out other causes of stroke in young patients, even when APS is suspected as part of the initial assessment. All risk factors for APS must be considered, and potential triggers should be identified. These patients require strict follow-up, beginning with confirmation of antiphospholipid antibodies 12 weeks after the clinical event. The relationship between infectious diseases and the onset of APS has been studied and documented, with infections acting as potential triggers through complex pathophysiological mechanisms. While identifying the cause of ischemic stroke in young adults and/or assessing APS, appropriate treatment must be initiated promptly to prevent complications. After the acute phase, patients require long-term management, primarily with appropriate anticoagulation, as part of their follow-up care.
